# Interference during the retrieval of arithmetic and lexico-semantic knowledge modulates similar brain regions: Evidence from functional magnetic resonance imaging (fMRI)

**DOI:** 10.1016/j.cortex.2019.06.007

**Published:** 2019-07-19

**Authors:** Alexander E. Heidekum, Roland H. Grabner, Bert De Smedt, Alice De Visscher, Stephan E. Vogel

**Affiliations:** a Educational Neuroscience, Institute of Psychology, University of Graz, Austria; b Faculty of Psychology and Educational Sciences, KU Leuven, University of Leuven, Belgium; c Psychological Sciences Research Institute, UCLouvain, Belgium

**Keywords:** Arithmetic fact, Inferior frontal gyrus, Interference, Intraparietal sulcus, Semantic control

## Abstract

Single-digit multiplications are mainly solved by memory retrieval. However, these problems are also prone to errors due to systematic interference (i.e., co-activation of interconnected but incorrect solutions). Semantic control processes are crucial to overcome this type of interference and to retrieve the correct information. Previous research suggests the importance of several brain regions such as the left inferior frontal cortex and the intraparietal sulcus (IPS) for semantic control. But, this evidence is mainly based on tasks measuring interference during the processing of lexico-semantic information (e.g., pictures or words). Here, we investigated whether semantic control during arithmetic problem solving (i.e., multiplication fact retrieval) draws upon similar or different brain mechanisms as in other semantic domains (i.e., lexico-semantic).

The brain activity of 46 students was measured with fMRI while participants performed an operand-related-lure (OR) and a picture-word (PW) task. In the OR task participants had to verify the correctness of a given solution to a single-digit multiplication. Similarly, in the PW task, participants had to judge whether a presented word matches the concept displayed in a picture or not.

Analyses showed that resolving interference in these two tasks modulates the activation of a widespread fronto-parietal network (e.g., left/right IFG, left insula lobe, left IPS). Importantly, conjunction analysis revealed a neural overlap in the left inferior frontal gyrus (IFG) pars triangularis and left IPS. Additional Bayesian analyses showed that regions that are thought to store lexico-semantic information (e.g., left middle temporal gyrus) did not show evidence for an arithmetic interference effect. Overall, our findings not only indicate that semantic control plays an important role in arithmetic problem solving but also that it is supported by common brain regions across semantic domains. Additionally, by conducting Bayesian analysis we confirmed the hypothesis that the semantic control network contributes differently to semantic tasks of various domains.

## 1 Introduction

Solving arithmetic problems swiftly and efficiently is a key cognitive competence in our daily life (Parsons & Bynner, 2005; Vogel & Grabner, 2015). The semantic knowledge of arithmetic facts provides a basic foundation of this competence and is typically engaged whenever we solve simple (single-digit) problems (Domahs & Delazer, 2005). This holds particularly true for single-digit multiplication problems such as 3 × 4 or 7 × 9 (Campbell & Xue, 2001). There is wide consensus that arithmetic facts are stored in an associative network in semantic memory, in which problems and their corresponding solutions are connected to each other (e.g., Ashcraft, 1987; Campbell, 1995; Lemaire & Siegler, 1995; McCloskey & Lindemann, 1992; Verguts & Fias, 2005). However, within this associative network, the presentation of a particular problem (e.g., 6 × 8) not only leads to the activation of its correct answer (i.e., 48), but also to a co-activation of incorrect solutions (e.g., 40, 56, 42, 54) related to associated problems (e.g., 5 × 8, 7 × 8, 6 × 7, 6 × 9). This co-activation of interconnected (incorrect) solutions causes interference that needs to be overcome by means of semantic control mechanisms in order to retrieve the correct answer.

An increasing number of neuroimaging studies have investigated the brain network that helps to overcome interference during semantic cognition (i.e., executive control processes that “ensure that the cognitive system generates representations and inferences that are suited to the immediate task or context”; Lambon Ralph, Jefferies, Patterson, & Rogers, 2016, p. 8). This research has revealed the importance of several brain areas in the frontal, temporal and parietal cortex, which are commonly labeled as semantic control network (SCN; for a recent review see Lambon Ralph et al., 2016). Together with domain-specific networks (i.e., brain regions associated with specialized knowledge structures; Spelke & Kinzler, 2007) the SCN supports the retrieval of semantic information — either by boosting weakly encoded information or by suppressing over-learned responses (Lambon Ralph et al., 2016; Noonan, Jefferies, Visser, & Lambon Ralph et al., 2013). While most of the conducted neuroimaging studies have explored the neural correlates of semantic control in the context of lexico-semantic knowledge (i.e., processing of pictures, words or objects; Whitney, Kirk, O'Sullivan, Lambon Ralph, & Jefferies, 2011), less focus has been given to better understand the neural correlates of semantic control during arithmetic fact retrieval. For instance, it is currently unclear to which extent the neural correlates of interference resolution during arithmetic fact retrieval draw upon similar or different brain regions in comparison to other semantic domains. The aim of the present functional magnetic resonance imaging (fMRI) study was to provide a first answer to this question.

There is increasing evidence from recent neuroimaging work that the neural correlate for the efficient retrieval of a solution to a given arithmetic problem lies in a network that comprises fronto-parietal brain regions (e.g., for a review see Menon, 2014). This network includes the left angular gyrus (AG), left hemispheric language areas, such as the inferior frontal gyrus (IFG), the middle (MTG) and superior temporal gyrus (STG), as well as the supramarginal gyrus (SMG; Klein, Moeller, Glauche, Weiller, & Willmes, 2013, 2014; Menon, 2014; Peters & De Smedt, 2018). Regions of this arithmetic network are typically engaged whenever we solve simple arithmetic problems (e.g., 2 × 8; 5 × 6) by directly retrieving the answer from memory (Delazer et al., 2003, 2005; Grabner et al., 2009; Ischebeck et al., 2006, 2009). However, arithmetic fact retrieval is also prone to errors that are not random. Indeed, false answers are frequently operand related (Campbell, 1994, 1997), i.e., solutions to problems differing by one operand (e.g., the answer 42^[6 × 7]^ to the problem 6 × 8). This pattern indicates that arithmetic fact retrieval is influenced by interferences that affect the correct retrieval of arithmetic solutions. A well-established task that is particularly suited to examine interference during arithmetic fact retrieval is the operand-related-lure (OR) task. In this task, single-digit multiplication problems are simultaneously presented with a correct or incorrect solution on a computer screen, and participants have to decide whether the presented solution is correct or not. Importantly, incorrect equations are divided into problems presented with a solution that is related to the correct result (operand-related-lure, belonging to the same multiplication table; e.g., 7 × 2 = 16^[8 × 2]^) or with a solution that is not related to the correct result (operand-unrelated-lure; e.g., 7 × 2 = 13). Behavioral studies have revealed an OR interference effect, consisting of lower accuracy and longer response times (RTs) in the operand-related compared to the operand-unrelated condition (e.g., Domahs et al., 2007; Zbrodoff & Logan, 1990). It has been argued that this behavioral effect reflects the associative interference characteristics of the arithmetic fact network as well as the cognitive resources (i.e., semantic control) that are needed to overcome them (Campbell, 1987; Zbrodoff & Logan, 1986).

To date, only a handful of studies have investigated the neural correlates of the OR interference effect. All of them have used event-related potentials (ERPs) to better understand the temporal brain dynamics associated with the interference effect (e.g., Domahs et al., 2007; Jost, Hennighausen, & Rösler, 2004; Niedeggen & Rösler, 1999). For instance, Niedeggen and Rösler (1999) provided evidence that the OR interference effect is associated with a negative brain potential in healthy adults, peaking between 300 and 500 msec. after the solution onset (i.e., thereafter called N400 effect). Furthermore, an amplitude difference was found in the late positive component (LPC), which was characterized by a positive peak between 540 and 620 msec. after solution onset. Whereas the difference in the LPC effect was present at all electrodes, the difference in the N400 effect was observed over posterior electrodes of the right hemisphere. The authors concluded that the electrophysiological response pattern of the N400 reflects the spreading activation (i.e., interference) within the arithmetic fact network, whereas the LPC reflects semantic control processes associated with overcoming interference. This finding provided first insights into the temporal dynamics of the interference effect during arithmetic fact retrieval. However, from this research it is not clear which brain regions were engaged and whether the brain mechanisms associated with processing different semantic information (e.g., words or pictures) map onto similar or different brain regions.

So far, only two neuroimaging studies have investigated neural interference effects during multiplication fact retrieval. In groups of healthy adults, De Visscher, Berens, Keidel, Noël, and Bird (2015, 2018) compared activation patterns of low and high interfering multiplication problems during problem-solving. The interference distinction was made based on the learning history in which multiplication facts are typically acquired (i.e., the order in which multiplication problems are taught in schools). Multiplications sharing lots of features (i.e., number of co-occurrences of digits) with previously learnt problems (e.g., 3 × 9 = 27 shares 3 digits with the previously encountered problem 3 × 7 = 21) were considered as high-interfering (e.g., 6 × 7 = 42), whereas those sharing fewer features were labeled low-interfering (e.g., 7 × 5 = 35). In both studies, participants were presented with single-digit multiplication problems followed by a solution option, which had to be indicated as correct or incorrect. The results of the first study showed that the verification of high-interfering multiplications was associated with greater brain activation in frontal regions [e.g., the left and right inferior frontal gyrus (IFG) and the insula lobes] compared to low-interfering multiplications. For the same contrast, the results of the second study revealed greater activation in parietal regions of the left intraparietal sulcus (IPS; De Visscher et al., 2018). The authors interpreted that these fronto-parietal activations are associated with a higher demand of cognitive control to process high-interfering problems compared to low-interfering problems. Interestingly, the location of these brain regions overlaps with areas that have been previously reported in studies investigating semantic control during the processing of lexico-semantic information (e.g., processing of words or pictures). However, the studies by De Visscher et al. (2015, 2018) aimed at investigating the interference effect associated with the learning history of multiplication tables. In their experimental design, the authors did not present a lure associated with the multiplication problem, but rather asked the participants to solve the problem in their mind (in maximum three seconds) and, afterwards, to verify a proposed answer. Therefore, these studies did not investigate the semantic interference triggered by an operand-related lure. In contrast to the design used by De Visscher et al. (2015, 2018), the OR task allows the direct induction of semantic interference by manipulating the semantic associations between multiplication problems and their correct and incorrect solutions. With this approach, differences in brain activation can be better linked to ongoing semantic control processes needed to overcome interference.

In contrast to the arithmetic domain, several studies have investigated the brain network that helps to overcome interference in other semantic domains, such as the naming of pictures. A task that has proven to be well suited to investigate semantic control mechanisms in the context of lexico-semantic processing is the picture-word (PW) task. In the PW task, participants are asked to name pictures while ignoring embedded and interfering distractors (i.e., words). Similar to the OR task, distractors belong to two different conditions: (1) related lures and (2) unrelated lures. Related lures are words belonging to the same semantic category as the picture (e.g., picture: *dog,* distractor word: *cat*) whereas unrelated lures do not (e.g., picture: *dog,* distractor word: *hat*). Similar to the interference effect observed in the OR task, naming pictures, while ignoring related lures, leads to a lexico-semantic interference effect associated with longer naming latencies and higher error rates compared to unrelated lures (e.g., Brown, 1981; Costa, Alario, & Caramazza, 2005; Damian & Bowers, 2003; de Zubicaray, Wilson, Mcmahon, & Muthiah, 2001; Glaser & Düngelhoff, 1984; Glaser & Glaser, 1989; Schriefers, Meyer, & Levelt, 1990). It is argued that prolonged RTs in the PW task reflect increased competition due to the activation of a semantic competitor (Spalek & Thompson-Schill, 2008).

Neuroimaging studies (e.g., de Zubicaray et al., 2001; Spalek & Thompson-Schill, 2008) focusing on the neural responses associated with the lexico-semantic interference effect in the PW task have consistently shown the involvement of frontal, temporal and parietal brain regions in the left hemisphere. For example, in a study by de Zubicaray et al. (2001), participants had to name pictures while ignoring the printed words which were simultaneously presented on a screen. Printed words were either from the same semantic category (semantically-related) or from a control condition with no semantic association (i.e., words were formed by a row of X's). De Zubicaray et al. (2001) reported differential hemodynamic response in the left mid-section of the MTG, the left posterior superior temporal gyrus (STG), the bilateral orbitomedial prefrontal cortex (PFC) and the left supramarginal gyrus (SMG) during the overt vocalization of picture names while ignoring words of the semantically-related condition compared to the control condition. Whereas activations in the mid-section of the MTG and posterior STG were related to the competition associated with the interference effect, frontal and parietal responses were interpreted as additional cognitive mechanisms responsible for interference resolution (i.e., interference detection and inhibitory control).

While the study by de Zubicaray et al. (2001) used a non-semantic control condition (i.e., a row of X's), a work by Abel et al. (2009) focused on more stringent contrasts between semantic-related and semantic-unrelated conditions. The authors presented distractor words over headphones to a group of adults. These distractor words preceded pictures presented on a screen and were drawn from following conditions: (1) distractor words that were from the same semantic category (e.g., picture: *candle,* distractor word: *lamp*), or (2) unrelated distractor words (e.g., distractor word: *kiwi*, picture: *bed*). By contrasting these two conditions the authors found that distractor words from the same semantic category lead to a higher activation in the left IFG pars orbitalis compared to words from an unrelated semantic category. The activation pattern in this neuroimaging study was interpreted to reflect control mechanisms that are associated with semantic retrieval and the processing of semantic relationships.

In their recent review, Lambon Ralph et al. (2016) summarize the results and emphasize the involvement of at least three different brain systems that underpin semantic representations and semantic control functions. The authors argued that multimodal experiential and language-supported representations are distributed over the entire cortex, supported by (a) the default mode network (DMN) and (b) a language-specific network that is located around the sylvian fissure (Xu, He, & Bi, 2017). These networks (for semantic representations) interact with a third and left lateralized executive control network (Lambon Ralph et al., 2016), which includes brain regions of the IFG (i.e., IFG pars opercularis, IFG pars triangularis, IFG pars orbitalis), the inferior frontal sulcus (IFS), the pre-supplementary motor area (pre-SMA), the posterior MTG, the AG and the IPS (Humphreys & Lambon Ralph, 2015; Lambon Ralph et al., 2016; Noonan et al., 2013; Xu et al., 2017). There is increasing evidence that the contribution of the SCN varies across different semantic tasks. For instance, Noonan et al. (2013) demonstrated that receptive (i.e., word/sentence comprehension) tasks modulate the brain activation of the posterior MTG, while no such modulation is found for production tasks (i.e., verb generation). This indicates that the engagement of the SCN is task dependent. Additionally, it is assumed that selective semantic retrieval not only relies on mechanisms specialized for the control of meaningful (semantic) associations but also on non-semantic (i.e., domain-general) control processes (e.g., Davey et al., 2016; Noonan et al., 2013). These non-semantic control processes are underpinned by cortical regions that usually respond to high cognitive demands across many tasks and that are located in the frontal and parietal cortex (often termed the frontoparietal control network or multiple demand network; e.g., Duncan, 2010; Power & Petersen, 2013). These brain regions include, among others, dorsal and posterior aspects of the IFS and the IPS (Lambon Ralph et al., 2016).

Against this background, two important conclusions can be drawn. First, interference effects arise in arithmetic fact retrieval and in the retrieval of lexico-semantic knowledge. In both domains, it is argued that interference emerges as a consequence of the way the information is stored, namely in an associative network. Second, in both domains control processes are needed to overcome associative interference in order to select the correct solution/response. However, the brain network supporting the required semantic control processes has predominantly been investigated for lexico-semantic knowledge. To the best of our knowledge, no neuroimaging study has investigated the brain regions associated with semantic control in the context of arithmetic fact retrieval. Therefore, it is currently unknown whether the activation patterns associated with semantic control are overlapping and/or distinct across these domains. Probing both conditions within one sample of subjects will provide novel evidence on the neural correlates associated with interference and semantic control.

In the present study, the OR and PW tasks were administered within a sample of healthy adults. The discussed similarities between the PW and the OR task make these two tasks ideal candidates to investigate semantic control mechanisms across different domains. Nevertheless, both tasks differ in response type that has to be given by the subjects – namely picture naming in the PW task and manual response selection (i.e., button press) in the OR task. Thus, to keep both paradigms comparable, we adapted the PW paradigm so it could be used as a verification task in the present work (the typical pattern of interference was confirmed in a pilot study previous to the fMRI study). In our adapted version pictures and words were presented simultaneously and subjects had to decide whether the meaning of the word matched the concept displayed on the picture or not. We argued that if overcoming interference during arithmetic fact retrieval relies on similar semantic control mechanisms as during the processing of words and pictures, we should find similar activation differences in cortical regions associated with the SCN when contrasting related with unrelated multiplication problems (hypothesis 1). Further, the involvement of the SCN should also be observed during overcoming lexico-semantic interference (hypothesis 2). As such, a significant overlap between brain activation patterns should be observed in regions associated with domain-general control mechanisms (hypothesis 3). And finally, if there are task-specific aspects contributing to the interference effects, we expect to observe an arithmetic interference effect in the absence of a lexico-semantic interference effect in cortical regions associated with arithmetic fact retrieval (e.g., AG), as well as a lexico-semantic interference effect in absence of an arithmetic interference effect in brain regions associated with the recognition of words and objects (e.g., areas in the temporal lobe; hypothesis 4).

## 2 Method

We report how we determined our sample size, all data exclusions, all inclusion/exclusion criteria, whether inclusion/exclusion criteria were established prior to data analysis, all manipulations, and all measures in the study. Furthermore, individual anonymized data and digital study materials (i.e., stimuli and experimental presentation code) can be accessed via the internet (https://osf.io/m6p82/, Heidekum, 2019).

### 2.1 Participants

Forty-six right-handed native-German-speaking students (29 females; mean age = 23.6, age range = 18–32) of the University of Graz participated in the present event-related functional magnetic resonance imaging (fMRI) study. Specification of the sample size was based on time constraints and available resources (e.g., money). Neither a history of psychiatric or neurological disorders nor a current use of psychoactive medications was reported by the participants, otherwise they were excluded from the study. All participants had normal or corrected to normal vision, provided informed consent and were compensated with a total of € 20 for 2 h of participation. The experimental procedure of the study was approved by the ethics committee at the University of Graz, Austria.

### 2.2 Materials and stimuli

To investigate task related commonalities and differences in the neural correlates associated with interference in memory retrieval and semantic control, participants performed two different verification tasks in the MRI scanner. The OR task (e.g., Domahs et al., 2007) was used to investigate brain activation associated with arithmetic interference and an adjusted version of the PW task (e.g., de Zubicaray, McMahon, Eastburn, & Wilson, 2002) was used to investigate the brain activation associated with lexico-semantic interference. Additionally, participants were given a third task (Winkelman & Schmidt, 1974) which was not further investigated in this study. The order of the tasks was counterbalanced between participants. Neither the study, nor the analyses were pre-registered.

#### 2.2.1 Operand-related-lure

In the OR task, single-digit multiplication problems with a correct or incorrect solution were presented on a computer screen. Participants had to decide whether the presented solution was correct or not. The set of problems in the OR task consisted of 72 single-digit multiplication problems (from 2 × 2 to 9 × 9, including tie problems such as 4 × 4; see Appendix Table A.1). Operands 0 and 1 were not used in order to exclude rule-based solving mechanisms reported in other studies (e.g., Jost, Beinhoff, Hennighausen, & Rösler, 2004). Multiplication equations were presented either with a correct or an incorrect solution. Importantly, for each problem two different incorrect solutions were constructed: Related and unrelated lures. Related lures were solutions from another but operand related multiplication problem (e.g., 2 × 7 = 16^[2 × 8]^). Only multiples of one of the problem's operands were used (i.e., m x n ± 1; e.g., 2 × 8 in the multiplication problem above). Unrelated lures were solutions not belonging to any conventional multiplication table (e.g., 8 × 4 = 26). The following confounds were kept constant: Lures were matched for distance to the correct result (because lures with a larger split are typically easier to reject; Zbrodoff & Logan, 1990). A paired sample *t*-test showed no significant difference in the distance to the correct result between related and unrelated lures (related lures: *M* = −.056, *SEM* = .815; unrelated lures: *M* = −.583, *SEM* = .786; *t*_35_ = −1.296, *p* = .203). Related and unrelated lures were matched for parity (Krueger, 1986) and decade (Domahs et al., 2007).

#### 2.2.2 Picture-word

**Fig. 1:**
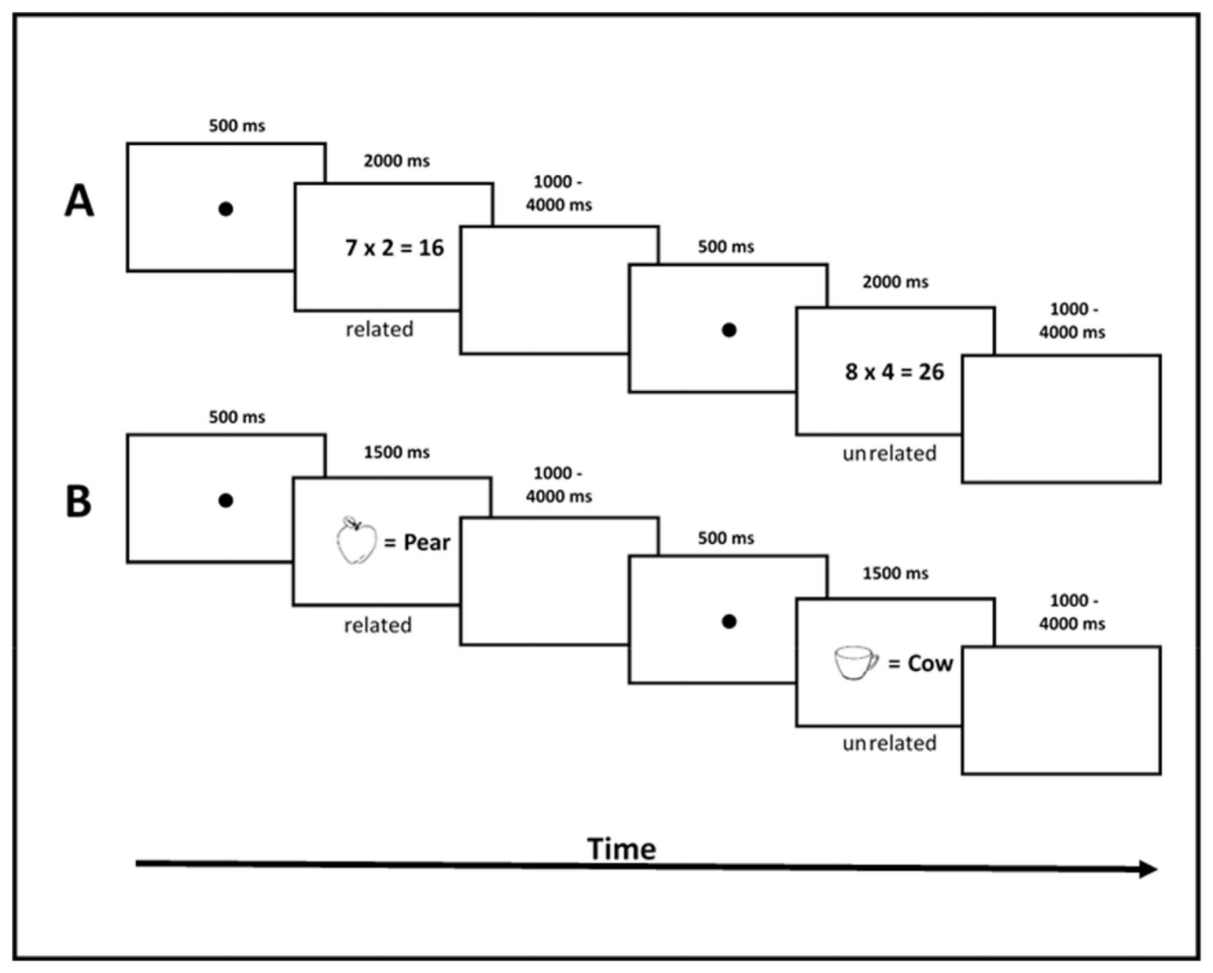
Time-course of two trials in (A) the operand-related-lure (OR) and (B) the picture-word (PW) task.

Picture-word pairs were presented on a computer screen. Participants had to judge whether the meaning of the word matched the concept displayed on the picture or not. In total, 32 black and white line-drawings from a standardized picture set (Snodgrass & Vanderwart, 1980) were used. The pictures illustrated 32 different one-word concepts (see Appendix Table A.2) of six semantic categories (i.e., animals, insects, plants, fruits, tools and clothing) adopted from Postler et al. (2003). Each picture was presented simultaneously with an equal sign and a written German word (see Fig. 1). Similar to the OR task, each picture was used in three different conditions: (1) the picture was presented with its correct word; (2) the picture and the incorrect word were from the same semantic category (e.g., picture: *dog*, distractor word: *cat;* related lure); (3) the picture was associated with a word of another semantic category (e.g., picture: *cup,* distractor word: *cat*; unrelated lure). Stimulus material of the three conditions were matched in written and spoken word frequency, in length (number of phonemes and graphemes), in visual complexity and in name agreement in percent (i.e., percentage of participants that gave the item the same name; see also Postler et al., 2003, for the statistical evaluation of the stimulus material).

### 2.3 Experimental procedure

Stimuli of both tasks were presented with PsychoPy (version 1.83.4; Peirce, 2007, 2008) on a 32″ Full HD LCD-Monitor situated behind the MRI scanner. Participants watched the presentation of the stimuli via a mounted mirror device on the head coil. The OR task took about 12 min and consisted of 4 runs. The whole stimulus set was presented twice, resulting in 144 trials overall. In every run, half of the multiplication equations were presented with their correct solution and the other half with an incorrect solution (half related lures, half unrelated lures). In the PW task (duration ca. 10 min), each black and white line-drawing (i.e., 32 pictures) was presented four times, resulting in 128 picture-word pairs. The task was divided into 4 runs each consisting of 32 problems. In half of the trials, the pictures were associated with their correct word (i.e., correct condition) and, in the other half, (i.e., incorrect condition) with a semantic related or unrelated lure (lures were evenly distributed).

Every trial of the two tasks started with 500 msec. fixation followed by a multiplication problem (in the OR task) or a picture-word pair (in the PW task). While problems were presented for 2000 msec. in the OR task, problems were presented for 1500 msec. (see Fig. 1) in the PW task. Participants were required to give the answer as accurate and as fast as possible. A jittered inter-stimulus-interval (blank screen) with a mean duration of 2500 msec. (1000–4000 msec.) was interspersed between trials. In both tasks, problems were presented following a pseudo-random order so that no more than three successive problems were of the same type (e.g., lure type, smaller operand first, larger operand first, or tie problem) and two successive problems never shared the same operands/concepts or the same answer.

### 2.4 MRI acquisition

Structural and functional imaging data were collected with a 3.0 T Siemens Skyra MRI scanner using a 32-channel head coil at the MRI Lab Graz. The functional images were obtained with a single shot gradient echo-planar imaging (EPI) sequence sensitive to blood oxygenation level dependent (BOLD) contrast [repetition time (TR) = 2530 msec., echo time (TE) = 36.4 msec., flip angle = 60°, field of view (FOV) = 215 mm]. In total, 52 transverse slices with a 2.5 × 2.5 × 2.5 mm isotropic voxel resolution were acquired with a multi-band sequence (acceleration factor of 4) in interleaved order parallel to the anterior commissure – posterior commissure (AC-PC) line. An average of 413 (*SD* = 6.7) functional images were collected during the OR task and an average of 344 (*SD* = 10.6) images were collected during the PW task. In addition, a high-resolution T1-weighted anatomical image of participant's brain was acquired with a Generalized Autocalibrating Partially Parallel Acquisitions (GRAPPA) sequence (TR = 1950 msec., TE = 2.89 msec., 1 × 1 × 1 mm isotropic voxel resolution). Finally, diffusion-weighted images (DWI) were acquired but they were not analyzed in context of the present study.

### 2.5 Analysis of behavioral data

The first analysis aimed to investigate the presence of the behavioral interference effects in both tasks (i.e., OR: arithmetic interference; PW: lexico-semantic interference). Therefore, mean response times (RT) and mean accuracy (ACC; see Table 1) were entered into two separate 2 (task: OR *vs* PW) × 3 (condition: correct *vs* related *vs* unrelated) Analyses of Variance (ANOVA). Only correctly solved trials were used to analyze the RT data. Simple effect analyses adjusted for multiple comparisons (Bonferroni) were calculated to check for significant main and interaction effects.

To examine differences in the size of the interference effects of the OR and PW task, individual interference values for each participant were computed using the formulas below. These interference values were then entered into two paired samples *t*-tests – one for ACC and one for RT.

(1) $${\text{Interference}\;\text{Value}}_{\text{ACC}}=\left[\left(\text{unrelated}\;\text{ACC-related}\;\text{ACC}\right)/\text{unrealated}\;\text{ACC}\right]*100$$

(2) $${\text{Interference}\;\text{Value}}_{\text{RT}}=\left[\left(\text{related}\;\text{RT-unrelated}\;\text{RT}\right)/\text{unrealated}\;\text{RT}\right]*100$$

### 2.6 Imaging preprocessing

Preprocessing of the imaging data was performed using the Data Processing Assistant for Resting-State fMRI (DPARSF, Yan & Zang, 2010, http://rfmri.org/DPARSF), which is based on the Statistical Parametric Mapping (SPM, Welcome Department of Imaging Neuroscience, London, U.K.) software and the toolbox for Data Processing & Analysis of Brain Imaging (DPABI, Yan, Wang, Zuo, & Zang, 2016, http://rmfri.org/DPABI). Functional images were first slice time corrected (referenced to the slice acquired at the middle time point) and corrected for motion (realignment). In a next step, the obtained structural images were co-registered to the functional space. The toolbox for “Diffeomorphic Anatomical Registration using Exponentiated Lie algebra” (DARTEL; Ashburner, 2007) was used to spatially transform images into standard space of the Montreal Neurological Institute (MNI). Finally, a 8.0 mm full-width-at-half-maximum (FWHM) Gaussian kernel was used to smooth the functional imaging data. Statistical analyzes were performed using SPM12. A general linear model (GLM) was calculated entering only correctly solved problems of each task conditions (i.e., OR correct, OR related, OR unrelated, PW correct, PW related, PW unrelated). Moreover, six motion parameters and errors for each task were entered as additional regressors of no interest. In order to remove low frequency modulations, a 128 sec high-pass filter was applied. Finally, to model the hemodynamic response, the predictors of each condition were convolved with SPM's canonical hemodynamic response function (HRF).

### 2.7 Functional data analysis

In two first whole brain analyses we investigated the brain regions that were associated with interference in each task (hypotheses 1 and 2). To investigate interference in the OR task, a first level contrast “related lure > unrelated lure” was calculated for each subject. This contrast reveals brain regions that show greater brain activation for the high interfering trials (operand-related answer) compared to the low interfering trials (unrelated answer). To investigate interference in the PW task, a first-level contrast “related lure > unrelated lure” was calculated for each subject. This contrast reveals brain regions that show greater activation for interfering trials (i.e., trials with a related semantic concept) compared to non-interfering trials (i.e., trials with an unrelated semantic concept). On the second level, the group data were analyzed with one sample *t*-tests to identify those brain regions associated with arithmetic and lexico-semantic interference.

The second whole brain analysis of the study aimed to unravel those brain regions that showed a significant overlap associated with interference effect in both tasks (hypothesis 3). For this, the same first level contrasts were used as in the previous analysis. On the second level, a conjunction analysis (Price & Friston, 1997) was performed on the group level. This analysis tested for regions that showed a significant activation in the “related lure > unrelated lure” contrast of the PW paradigm AND a significant activation in the “related lure > unrelated lure” contrast of the OR paradigm. The statistical results of these whole-brain analyses are reported with family wise error (FWE) corrected values at the peak level (*p* < .05).

Performing a conjunction analysis is the classical method to investigate communalities between cognitive mechanisms. However, this analysis does not allow us to answer the question, in which cortical regions activity is modulated only by one interference effect in absence of the second one (hypothesis 4). Furthermore, bayesian analysis has the advantage of mitigating the multiple comparisons problem because there is no need to correct for multiple testing (Dienes, 2011). Therefore, it increases the efficiency to detect subtle differences between conditions, which are masked on the whole brain level (Poldrack, 2007). Because of these advantages, we performed additional Bayesian analyses (Jarosz & Wiley, 2014; Wagenmakers, 2007) on the neuroimaging data [i.e., mean percent signal change (PSC)] extracted from functional regions of interests (ROIs) based on the first whole brain analysis (i.e., OR related > OR unrelated; PW related > PW unrelated). Bayesian analysis was performed with Jasp 0.8.6 (JASP Team, 2018) and was implemented, because it allows us to quantify the evidence that speaks in favor of the null hypothesis (i.e., the likelihood of no interference effect in a particular ROI; Jarosz & Wiley, 2014). According to Jeffreys (1961), Bayes factors (BF_01_) of 1–3, 3–10, 10–30, 30–100, >150 respectively point towards anecdotal, substantial, strong, very strong or decisive evidence for the null hypothesis. Additionally, Bayesian analysis allows us to verify the extent to which the data are in favor of the alternative hypothesis (i.e., the likelihood of an interference effect in a particular ROI). Bayes factors (BF_01_) of 1–.33, .33–.10, .10–.03, .03–.001, <.001 respectively point towards anecdotal, substantial, strong, very strong, or decisive evidence for the alternative hypothesis (Jeffreys, 1961).

As a first step, we calculated BF_01_ for a neural lexico-semantic interference effect in functional ROIs that were found to be activated when contrasting related with unrelated multiplication problems. In the next step, we calculated BF_01_ for a neural arithmetic interference effect in functional ROIs associated with the neural lexico-semantic interference effect. The aim of these analyses were to unravel those brain regions that were modulated by one interference effect (e.g., PW interference) in absence of the second one (e.g., OR interference).

## 3 Results

### 3.1 Response latencies and accuracy

Results of the ANOVA on RTs revealed a main effect of task (*F*_1,45_ = 257.64, *p* < .001, η^2^ = .85), demonstrating a significant difference between mean RTs in the OR task (*M* = 1357 msec., *SEM* = 32 msec.) and those in the PW task (*M* = 948 msec., *SEM* = 16). There was also a significant main effect of condition (*F*_2,90_ = 170.03, *p* < .001, η^2^ = .79). Post-hoc simple effect analysis revealed that problems (multiplication equations and picture-word pairs) of the correct condition (*F*_2,44_ = 193.97, *p* < .001, *M* = 1091 msec., *SEM* = 19 msec.) were associated with shorter RTs compared to problems of the related (*F*_2,44_ = 193.97, *p* < .001, *M* = 1244 msec., *SEM* = 24 msec.) and unrelated condition (*F*_2,44_ = 193.97, *p* < .001, *M* = 1122 msec., *SEM* = 23 msec.). Importantly, problems of the related condition were significantly associated with longer response times than problems of the unrelated condition (i.e., interference effect; *F*_2,44_ = 193.97, *p* < .001). A significant interaction (*F*_2,90_ = 27.03, *p* < .001, η^2^ = .38) indicated that response times of the various conditions differed between both tasks. To unpack the observed task × condition interaction effect, we performed post-hoc simple effect analysis (see Table 1). This analysis revealed that in the OR task problems of the correct condition (*F*_2,44_ = 108.34, *p* < .001; *M* = 1266 msec., *SEM* = 29) were associated with shorter RTs compared to problems of the related (*F*_2,44_ = 108.34, *p* < .001; *M* = 1472 msec., *SEM* = 36) and unrelated condition (*F*_2,44_ = 108.34, *p* < .001; *M* = 1332 msec., *SEM* = 33). Additionally, RTs of problems of the related condition were significant longer compared to those of the unrelated condition (*F*_2,44_ = 108.34, *p* < .001). For the PW task, we found that problems of the related condition (*F*_2,44_ = 108.34, *p* < .001; *M* = 1017 msec., *SEM* = 17) were associated with longer RTs compared to problems of the correct (*F*_2,44_ = 108.34, *p* < .001; *M* = 917 msec., *SEM* = 15) and unrelated condition (*F*_2,44_ = 108.34, *p* < .001; *M* = 911 msec., *SEM* = 17). In contrast to the OR task, RTs between the correct and unrelated condition did not differ significantly. This difference in reaction time patterns explains the observed task × condition interaction.

A similar pattern of findings was observed for participants' accuracy data. The ANOVA revealed a significant main effect of task (*F*_1,45_ = 45.65, *p* < .001, η^2^ = .50), indicating that more errors were made in the OR task (*M* = .91, *SEM* = .007) compared to the PW task (*M* = .95, *SEM* = .003). There was also a significant main effect of condition (*F*_2,90_ = 155.07, *p* < .001, η^2^ = .78). Simple effect analysis revealed that significantly more errors were made in the related condition (*F*_2,44_ = 104.12, *p* < .001, *M* = .86, *SEM* = .008) compared to the correct (*F*_2,44_ = 104.12, *p* < .001, *M* = .96, *SEM* = .003) and the unrelated condition (*F*_2,44_ = 104.12, *p* < .001, *M* = .97, *SEM* = .005). No significant interaction was found (*F*_2,90_ = .84, *p* = .436).

Finally, additional analyses for comparing the individual interference values showed no significant difference in the sizes of interference values for the OR and the PW paradigm, neither for response times (*t*_45_ = .89, *p* = .380) nor for accuracy (*t*_45_ = −.41, *p* = .689). Thus, the size of the interference effects was comparable.

### 3.2 Task related brain activations of the interference effects

**Fig. 2:**
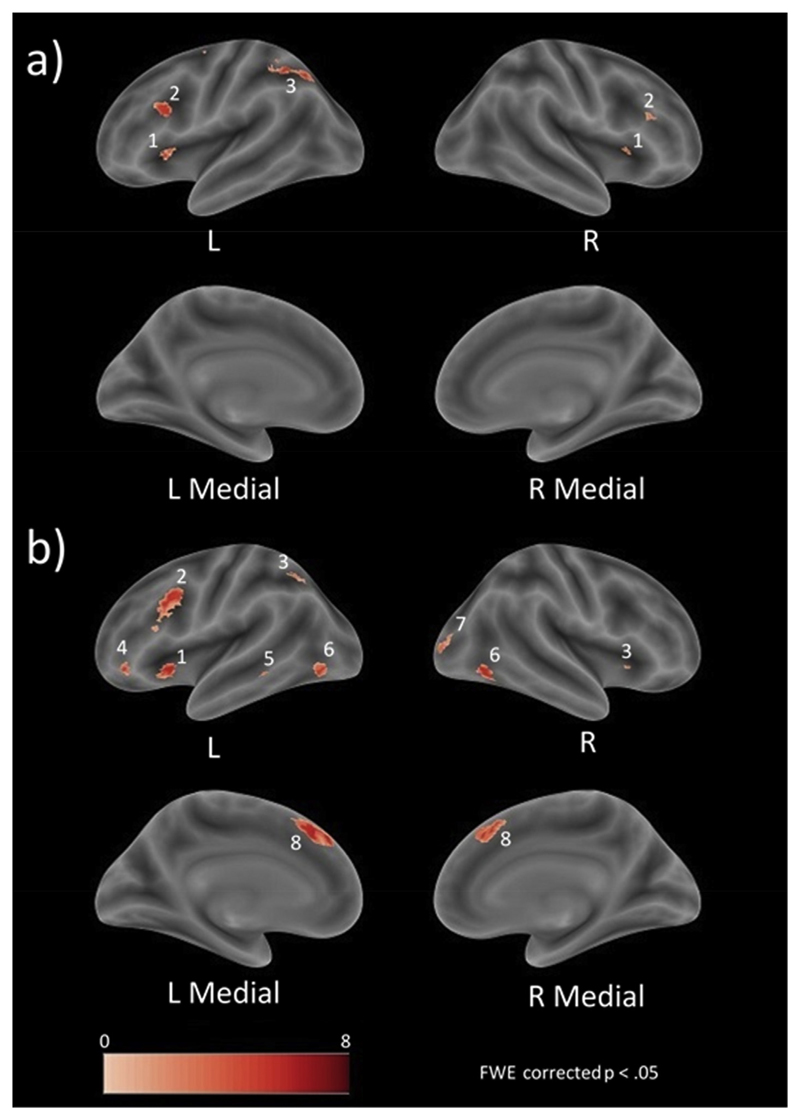
Brain regions associated with a) arithmetic interference (related > unrelated multiplication solutions) and b) lexico-semantic interference (related > unrelated picture-word pairs). (1) insula lobe, (2) IFG, (3) IPS, (4) IFG (p. Orbitalis), (5) MTG, (6) IFO, (7) middle occipital gyrus (MOG), (8) medial part of the SFG.

The first whole brain analyses investigated the brain regions that are associated with the interference effect in each task. The analysis on the OR task (i.e., arithmetic interference) revealed significant greater brain activations for related lures compared to unrelated lures in both hemispheres (see also Fig. 2, a or Table 2, a). More specifically, significant bilateral brain activation was observed in the insula lobe and the IFG. Brain regions of the left hemisphere included the superior frontal lobule (SFL) extending to the middle frontal gyrus (MFG) and the IPS. In the PW task (i.e., lexico-semantic interference), a number of significant brain activations in the left and right hemisphere (see also Fig. 2, b or Table 2, b) emerged for the same contrast (i.e., related lure > unrelated lure). More specifically, bilateral brain activations were observed in the IFG and the inferior occipital gyrus (IOG). Left hemispheric activations were found to be significant in the IPS and the MTG, whereas significant right hemispheric activation was observed in the medial part of the superior frontal gyrus (SFG) including the SMA.

### 3.3 Commonalities between both interference effects

**Fig. 3:**
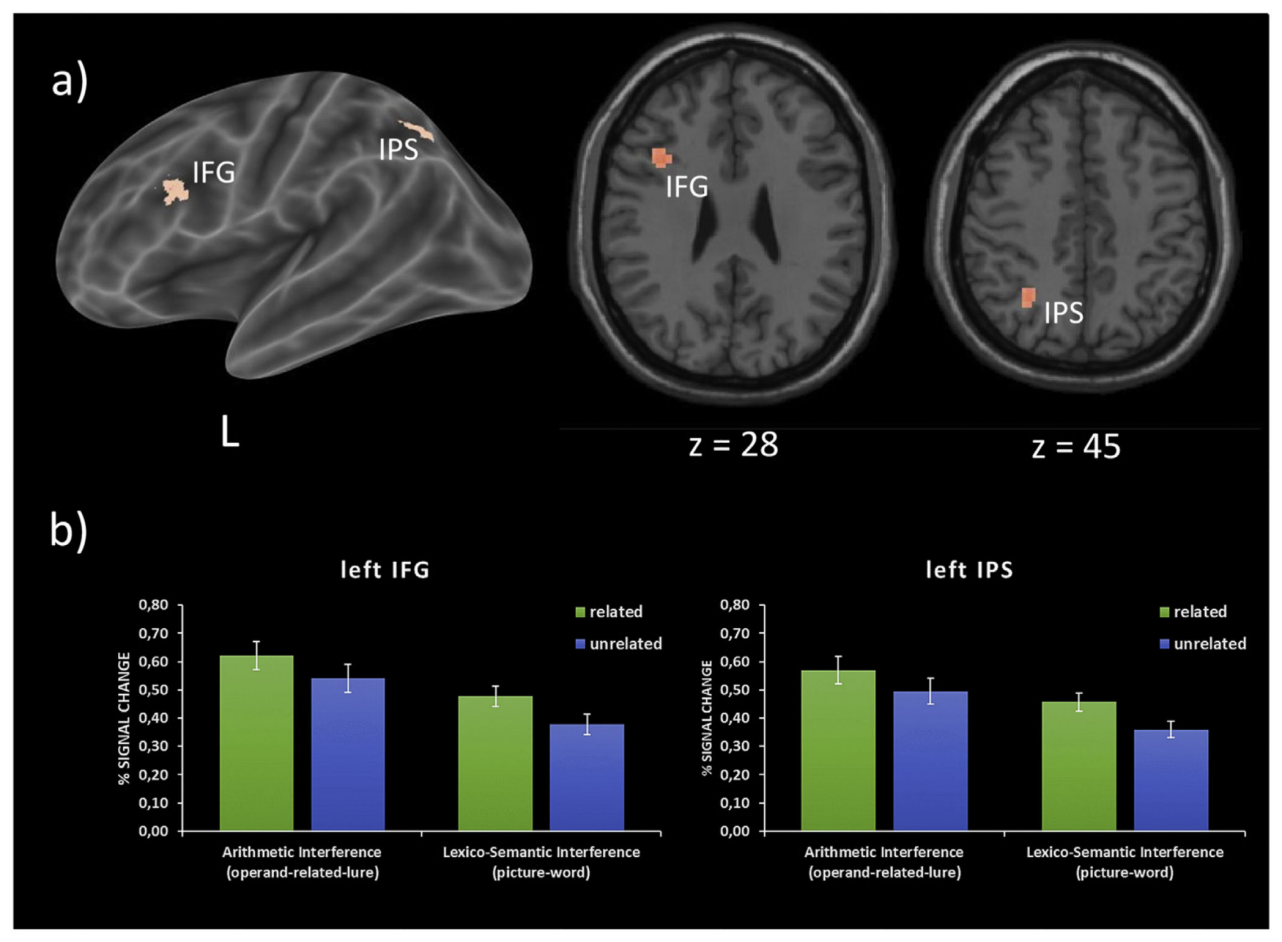
a) Brain regions showing a significant interference modulation in both tasks (i.e., conjunction between lexico-semantic and arithmetic interference). b) Plots showing percent signal change for each task (OR & PW) and each condition (related *vs* unrelated) separately for both brain regions found in the conjunction analysis.

To unravel those brain regions that showed a significant activation overlap across both interference effects, a conjunction analysis was computed. This analysis showed that the brain activation accompanying both lexico-semantic and arithmetic interference overlapped in two brain regions (see also Fig. 3a, b), i.e., the left IFG pars triangularis (MNI_(x,y,z)_: −38, 15, 28; t = 5.20; Z = 4.96; k = 31) and the left IPS (MNI_(x,y,z)_: −30, −58, 45; t = 5.12; Z = 4.88; k = 24). No other regions of this conjunction analysis showed a significant overlap of both interference effects.

### 3.4 Differences between both interference effects

**Fig. 4:**
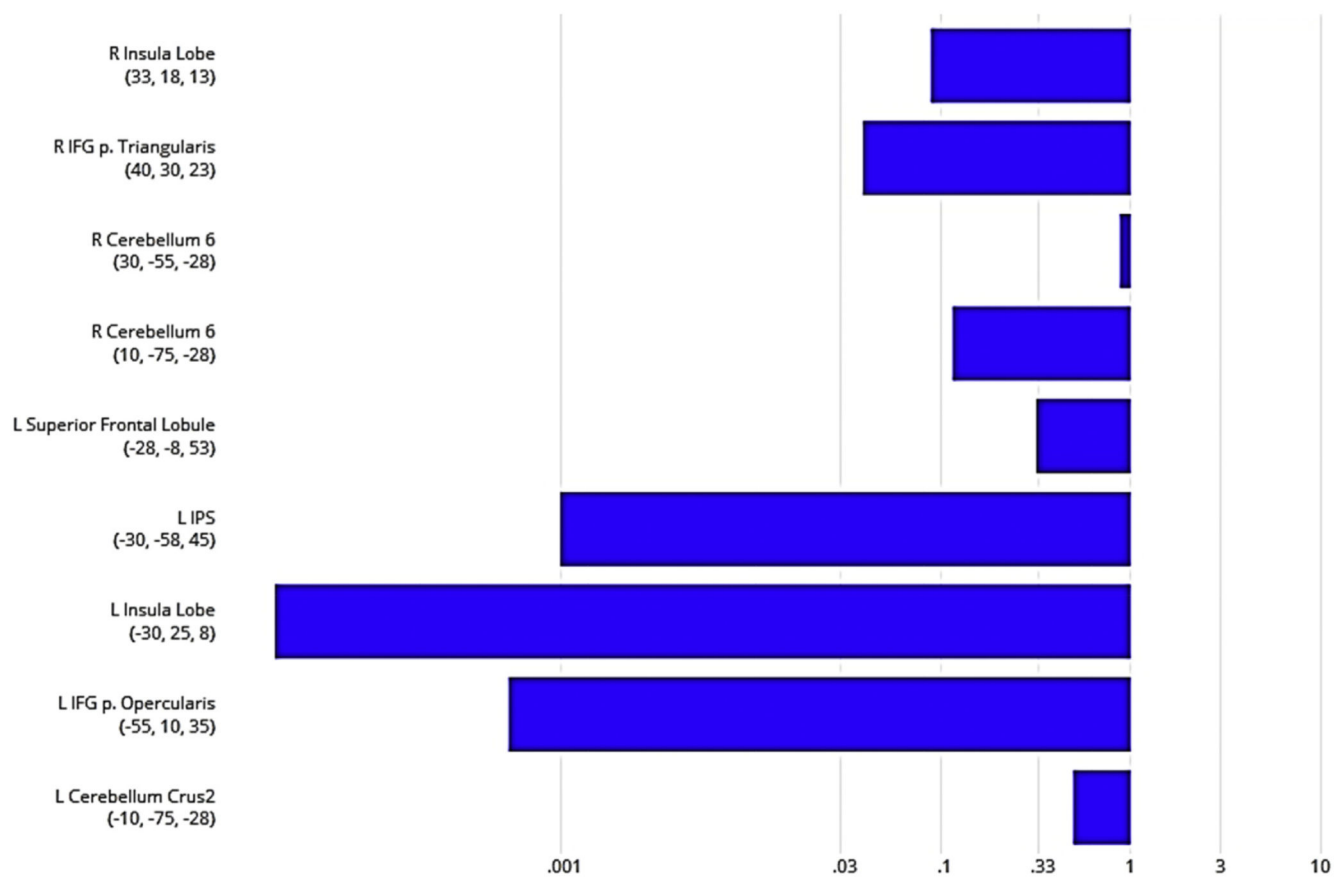
BF_01_ in favor of no lexico-semantic interference effect in brain regions associated with a neural arithmetic interference effect. Xaxis (log-scaled) showing BF_01_. Blue bars indicate more evidence against the null hypothesis of no effect, hence in favor of the hypothesis of an effect. Further, values smaller than .33 point towards substantial evidence for the hypothesis of an effect (interference effect). Green bars indicate evidence in favor of the null hypothesis of no effect. Values greater than 3 point towards substantial evidence for the null hypothesis of no effect (no interference effect).

Finally, Bayesian analyses were performed to reveal in which brain regions activity was modulated by one interference effect in absence of the second one. Firstly, we calculated BF_01_ in favor of a null effect for lexico-semantic interference (i.e., no BOLD difference between related and unrelated picture-word pairs) for each brain region identified in the first whole brain analysis (i.e., related lures > unrelated lures in the OR task; see Fig. 4). This analysis revealed for most of the brain regions no evidence for the null hypothesis, meaning that we found strong evidence for the alternative hypothesis (i.e., BF_01_ < .01). In other words, most of the brain regions that exhibited an arithmetic interference effect also showed a lexico-semantic interference effect.

**Fig. 5:**
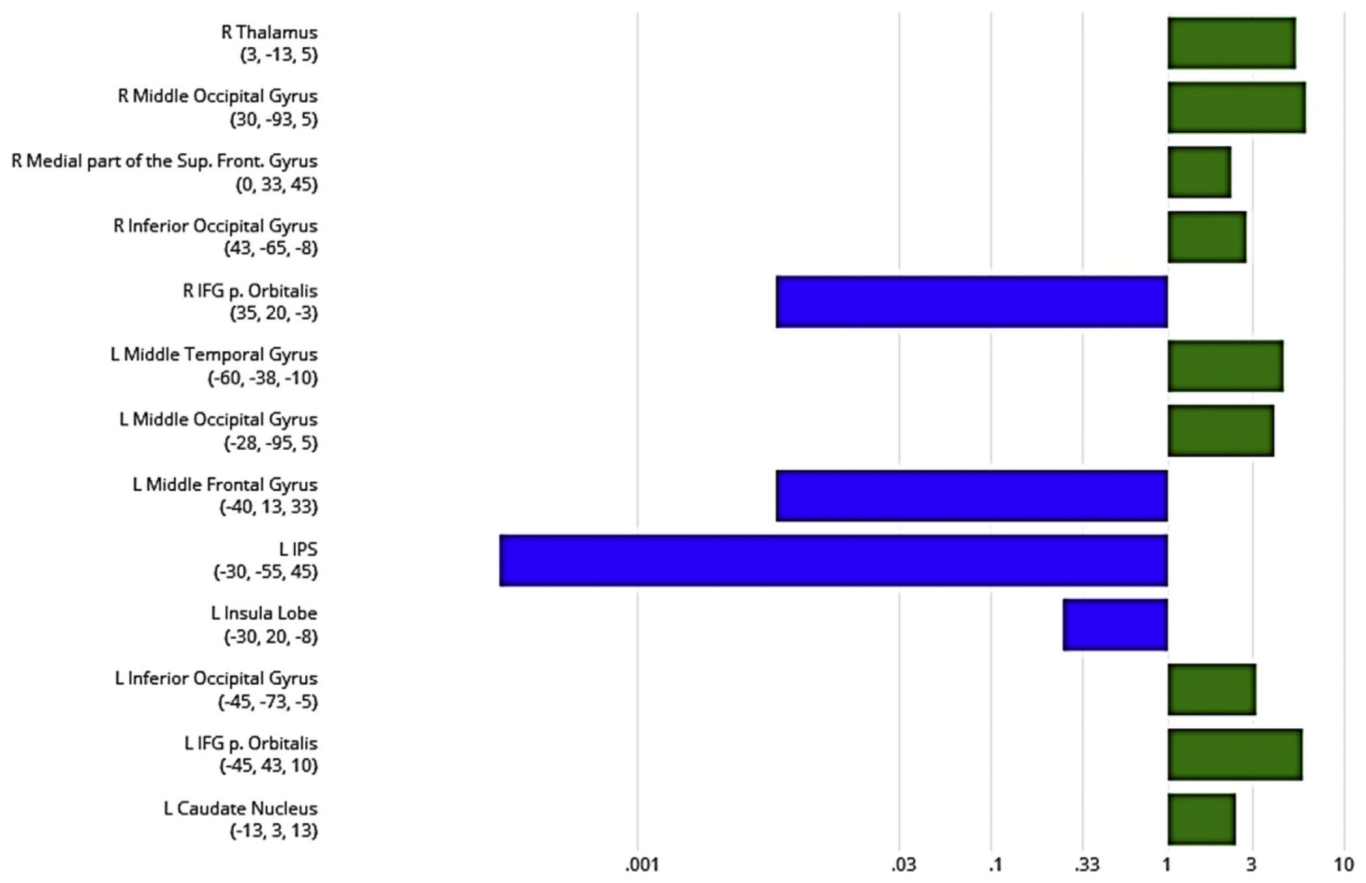
BF_01_ in favor of no arithmetic interference effect in brain regions associated with a neural lexico-semantic interference effect. Xaxis (log-scaled) showing BF_01_. Blue bars indicate more evidence against the null hypothesis of no effect, hence in favor of the hypothesis of an effect. Further, values smaller than .33 point towards substantial evidence for the hypothesis of an effect (interference effect). Green bars indicate evidence in favor of the null hypothesis of no effect. Values greater than 3 point towards substantial evidence for the null hypothesis of no effect (no interference effect).

Secondly, we calculated BF_01_ in favor of no arithmetic interference effect (i.e., no BOLD difference between related and unrelated multiplication problems) for brain regions identified in the second whole brain analysis (i.e., related lures > unrelated lures in the PW task; see Fig. 5). The values of BF_01_ suggested substantial evidence (i.e., BF_01_ > 3) that activity in the thalamus and the middle occipital gyrus (MOG) of the right hemisphere and activity in the left MOG, IFG (pars Orbitalis) and MTG was not modulated by the arithmetic interference effect. On the other hand, in the left insula lobe (MNI_(x,y,z)_: −30, 20, −8), the left IPS (MNI_(x,y,z)_: −30, −55, 45), the right IFG (pars orbitalis; MNI_(x,y,z)_: 35, 20, −3) and the left MFG (MNI_(x,y,z)_: −40, 13, 33), there was evidence for the presence of an arithmetic interference effect, indicating that these regions also showed an activation difference between related and unrelated multiplication problems.

## 4 Discussion

This study set out to better characterize the neural aspects of semantic control to overcome interference during multiplication fact retrieval. We investigated the question whether semantic control during multiplication fact retrieval draws upon similar and/or different brain mechanisms as in other semantic domains (i.e., retrieval of lexico-semantic information). In the present event-related fMRI study, we applied two well-established tasks to investigate this question. The OR task was used to study semantic control for resolving interference during the retrieval of multiplication facts, whereas an adapted version of the PW task was used to investigate semantic control during picture-word comparison.

### 4.1 Behavioral findings related to interference

In line with previous studies (e.g., Domahs et al., 2007; Jost, Hennighausen, et al., 2004; Niedeggen & Rösler, 1999), we found a significant behavioral interference effect (i.e., longer RTs and higher error rates for related compared to unrelated stimuli) in both the OR and PW task. Previous work (e.g., Campbell, 1987; Duncan-Johnson & Kopell, 1981) has suggested that these differences in RTs (i.e., behavioral interference effects) are due to response competitions arising after the activation of interconnected but false answers. Specifically, the higher decision time in the related condition compared to the unrelated condition is thought to reflect the additional cognitive effort to inhibit the competing response which is triggered by the stronger activation of the related lure. This explanation accounts for the arithmetic interference effect as well as for the lexico-semantic interference effect.

Referring to the behavioral results of the PW task, it should be highlighted that previous studies (e.g., Alario, Segui, & Ferrand, 2000; Costa et al., 2005; Damian & Bowers, 2003; Glaser & Düngelhoff, 1984) assessed lexico-interference by mainly using picture naming instead of a verification task. These studies showed that semantically related distractor words, embedded within a picture, slow down picture naming responses (i.e., interference effect). In line with this finding, we observed an interference effect of semantically related words on manual response selection (i.e., button press) after picture-word comparison. To the best of our knowledge, only one other study (Lupker & Katz, 1981) has ever observed the same effect by using a PW verification task to date.

Importantly for the subsequent interpretations of brain activation, the size of the arithmetic and lexico-semantic interference effect showed no significant difference (i.e., the reaction time differences between the interference effects did not differ). Accordingly, activation differences cannot be attributed to differences in the size of the interference effect (e.g., one interference effect being larger/smallerthan the other).

### 4.2 Neural correlates related to arithmetic and lexico-semantic interference

Firstly, we found that interference during the rejection of related multiplication problems in the OR task led to an activation of a widespread cortical network including the insula lobes, the IFG, the SFL, extending to the MFG, and the IPS of the left hemisphere. Similar regions were reported by a meta-analysis conducted by Arsalidou and Taylor (2011). In this study the authors identified brain regions that were involved in a variety of number and calculation (i.e., additions, multiplications and subtractions) tasks. They found that in addition to other cortical regions the left and right IFG and the MFG were commonly activated during multiplication problem solving. However, the underlying studies of this meta-analysis did not distinguish between problems requiring high and low semantic control. In contrast to these studies, De Visscher et al. (2015, 2018) differentiated between high and low interfering problems. In line with our results, they showed that high interfering multiplications activated the left/right IFG, left/right insula lobe and the left IPS (among other regions). They concluded that these activations reflect ongoing cognitive control processes that support the retrieval of arithmetic facts; an assumption that is in accordance with the literature of semantic cognition.

Secondly, we observed a neural lexico-semantic interference effect in the PW task. Greater brain activation was found in the left and right IFG, the left MTG and in the left IPS when related picture-word pairs were contrasted with unrelated picture-word pairs. This finding is in line with previous neuroimaging work in which similar neural lexico-semantic interference effects were found (e.g., de Zubicaray et al., 2001; Spalek & Thompson-Schill, 2008). Importantly, the cortical regions of our study also overlap with brain areas that were recently identified in a large-scale meta-analysis contacted by Noonan et al. (2013). The authors examined 53 neuroimaging studies that contrasted semantic tasks with high versus low control demands. The results of this work provided strong evidence that the left and right IFG (i.e., IFG pars opercularis, IFG pars triangularis, and IFG pars orbitalis), the right insula and parts of the left MTG are associated with selective semantic retrieval. For instance, an increase in BOLD response within the IFG is linked to controlled access to stored conceptual representations and processes that aim to select task-relevant representations from competing alternatives (Badre & Wagner, 2007). Thus, higher activation within the left IFG during the rejection of false but related picture-word pairs could reflect the active selection process that helps to generate the correct response and, therefore, dismisses the proposed related false response. An assumption that should also account for observed activation in the IFG during the processing of related multiplication problems. However, observed activation patterns for both tasks suggest the involvement of additional multiple demand control mechanisms to resolve interference elicited by the processing of related problems (i.e., multiplications and picture-word pairs).

### 4.3 Neural overlap between both interference effects

In order to explore whether semantic control during multiplication fact retrieval draws upon similar brain regions as during the retrieval of lexico-semantic information, we conducted a conjunction analysis. This analysis revealed that both interference effects showed an overlap in the frontal (i.e., IFG) and parietal lobe (i.e., IPS). Consistent with the task specific analyses described above the results identified a significant cluster in the IFG (MNI_(x,y,z)_: −38, 15, 28). This significant conjunction indicates that cognitive processes associated with lexico-semantic and arithmetic interference recruit similar brain regions. One possibility that could be associated with this joint activation is that the semantic control mechanisms, as described in the section above, drives this co-activation. Nevertheless, there is also the possibility that the identified brain activation is associated to other, more domain general computations. Indeed, the coordinates of the conjunction analysis are in close vicinity to the inferior frontal sulcus (IFS), which has previously reported to be part of a unitary system that contributes to executive control. This system is often termed the frontoparietal control network or multiple demand network (e.g., Duncan, 2010; Power & Petersen, 2013) and it is assumed that this system initiates and adapts control on a trial-by-trial basis (Dosenbach et al., 2006, 2007), such as response selection, inhibitory control (Chen, Lei, Ding, Li, & Chen, 2013; Hampshire & Sharp, 2015) and attentional orientation to task-relevant information (Corbetta & Shulman, 2002). In the present work a clear distinction between the mechanisms associated with the IFG and IFS is not possible. This is especially true since there are no clear-cut borders between these regions and its brain function but rather gradient distributions of these mechanism and the underlying neural substrate (Lambon Ralph et al., 2016). A distinction between the associated functions is further complicated by evidence that has shown that the multiple demand network (i.e., IFS and IPS) interacts with brain regions associated with the semantic control network (e.g., IFG or pMTG), especially during the controlled retrieval of meaningful associations (e.g., Davey et al., 2016; Noonan et al., 2013). As such, it highlights the importance for further research to clarify the involvement of the IFG and IFS and to disentangle the specific contribution of their associated mental functions during semantic interference control.

One argument that speaks in favor for the engagement of the multiple demand network, is the common involvement of the IPS (MNI_(x,y,z)_: −38, 15, 28) in both tasks. Previous work has shown that this cortical region might be also part of the multiple demand system (e.g., Duncan, 2010). Comparable to the IFS, the IPS is thought to serve a variety of control functions that generalize across different domains and tasks (e.g., Corbetta & Shulman, 2002; Scolari, Seidl-Rathkopf, & Kastner, 2015). Furthermore, the observed activation overlap in the left IPS is of particular interest because previous studies in the field of mathematical cognition related brain activity in this cortical region to the representation of numerical magnitude (Dehaene, Piazza, Pinel, & Cohen, 2003; or for a recent meta-analysis see; Sokolowski, Fias, Mousa, & Ansari, 2017). For instance, increased activation of the IPS is typically observed during number comparison in which subjects explicitly or implicitly compare the magnitude of numerals or dot-arrays (e.g., Ansari, Garcia, Lucas, Hamon, & Dhitalm, 2005; Pinel, Dehaene, Rivière, & LeBihan, 2001; Vogel et al., 2017; Wilkey, Barone, Mazzocco, Vogel, & Price, 2017). Additionally, the IPS was also found to be involved whenever procedural strategies are used to solve complex arithmetic problems. This brain activation is typically related to the manipulation of numerical magnitudes, which is needed to solve arithmetic problems (e.g., Delazer et al., 2003; Grabner et al., 2009). However, this domain-specific interpretation (i.e., quantity manipulation) of IPS activation was questioned by Fias, Menon, and Szucs (2013). By reviewing the existing literature, they showed that the IPS is not only involved during number processing but contributes also to basic neurocognitive functions, such as working memory (nevertheless, see also studies that have found significant brain activation in response to numerical stimuli during passive tasks in which the cognitive load is highly reduced, e.g., Vogel et al., 2017, Vogel and Grabner, 2015). Conversely, the current study provided novel evidence for an engagement of the left IPS in semantic control processes. Cognitive processes that are needed to resolve interference during memory retrieval. This finding is in line with the assumption (Ciaramelli, Gradi, & Moscovitch, 2008) that the IPS is involved whenever pre- or post-retrieval processes are required to support decision-making based on memory. In particular, Ciaramelli, Gradi and Moscovitch (2008) found that activity in the IPS increases whenever there are higher demands on memory search and monitoring, resulting from uncertainty in the retrieved memory products. For example, higher activation within the IPS is observed when there is a high similarity between studied targets and lures (i.e., related lures; e.g., Yago & Ishai, 2006). In the present study, higher activation in the left IPS for related compared to unrelated lures (i.e., multiplications and picture-word pairs) was observed, which might reflect the higher requirement for additional monitoring as a result of the association between related lures and correct answers. Nonetheless, statistical overlap in brain activation revealed by a conjunction analysis does not necessarily indicate that both tasks draw upon similar processing mechanisms. Thus, for future studies we suggest the use of multivariate methods, such as representational similarity analysis (RSA; Kriegeskorte, Mur, & Bandettini, 2008), to better address this question. Moreover, future research should try to disentangle IPS' brain activation associated with magnitude processing and cognitive control (e.g., Matejko & Ansari, 2017).

### 4.4 Differences between both interference effects

Finally, we performed Bayesian analyses to investigate, in which cortical regions activity was modulated only by one interference effect (e.g., the OR interference effect) in absence of the second one (e.g., the PW interference effect). In regions associated with an arithmetic interference effect, data provided no evidence in favor of the null hypothesis (i.e., no lexico-semantic interference effect). This suggests that these cortical regions (e.g., left and right insula lobe and IFG, left SFL/MFL and IPS) showed both an OR and a PW effect. Further, by investigating brain regions that showed a lexico-semantic interference effect, we found substantial evidence for the absence of a neural arithmetic interference effect in the thalamus and the middle occipital gyrus (MOG) of the right hemisphere and in the left MOG, IFG (pars orbitalis) and middle temporal gyrus (MTG). This indicates that these regions were modulated only by the PW effect. This result is in line with findings by Noonan et al. (2013), which provided evidence that the SCN is engaged differently across various semantic tasks. For instance, Noonan et al. (2013) demonstrated that receptive (i.e., word/sentence comprehension) tasks modulate the brain activation of the posterior MTG, while no such modulation is found for production tasks (i.e., verb generation). Here, we found a lexico-semantic interference effect in the absence of an arithmetic interference effect in brain regions that are thought to be involved in the semantic representation of objects (e.g., Bar et al., 2006; Malach, Levy, & Hasson, 2002). While arithmetic facts are thought to be stored as verbal codes in semantic memory (Dehaene et al., 2003), the PW task also required the processing of additional visual information (i.e., pictures of objects and animals), which could explain the involvement of the above-mentioned brain regions of the ventral stream. In contrast to this finding, we observe evidence in favor of an arithmetic interference effect in the left insular lobe, the left IPS, the right IFG and the left MFG, which partially overlaps with the result of the conjunction analysis.

### 4.5 Summary and suggestions for future research

Taken together, the present study provides evidence for the involvement of brain regions associated with semantic control processes during arithmetic fact retrieval (i.e., left IFG). These semantic control processes are underpinned by a widespread fronto-parietal brain network that has been shown to be involved in resolving interference during the retrieval of lexico-semantic information (i.e., pictures and words). Further, by using classical conjunction analysis we showed that activation patterns associated with the controlled retrieval of arithmetic facts and lexico-semantic information overlap in the left IFG pars triangularis and the left IPS. This finding suggests that both cortical regions are involved in interference resolution as part of a domain-general control network (i.e., multiple demand network) that interacts with brain regions associated with controlled semantic retrieval. Finally, Bayesian analyses revealed that cortical regions that are thought to store lexico-semantic information (e.g., left MTG) did not show evidence for an arithmetic interference effect. Thus, our results confirmed the assumption that the SCN contributes differently to semantic tasks of various domains (Noonan et al., 2013).

Nevertheless, there is an important aspect future studies should account for: It has been suggested that individual differences in interference effects may play an important role when learning arithmetic problems. More precisely, De Visscher and Noël (2014) proposed that a hypersensitivity to interference (i.e., less capacity to overcome interference) could prevent the development of an adequate arithmetic fact network because of the representational overlap of multiplication facts (in terms of shared digit associations). Interestingly, in an fMRI study De Visscher et al. (2018) observed a relationship between a neural interference effect (activation differences between interfering and non-interfering multiplication problems) and simple arithmetic performance in the left IFG. Specifically, they found that individuals with low arithmetic abilities showed a higher interference effect in this brain region. Based on this finding impaired semantic control processes could be related to hypersensitivity to interference. However, the current study did not aim to investigate individual differences in semantic control and its impact on overcoming interference during the retrieval of arithmetic facts. For that reason, future studies should investigate semantic control in groups with low and high arithmetic competencies and the relationship between the intrinsic connectivity of the SCN and various mathematical abilities (e.g., number processing, arithmetic, higher-order mathematics).

## Supplementary Material

Appendix A

## Figures and Tables

**Table 1:** Mean response times and mean accuracy for the lexico-semantic and the arithmetic interference task.

Task	Correct		Related		Unrelated	
	Mean RT	Accuracy	Mean RT	Accuracy	Mean RT	Accuracy
Lexico-semantic Interference (picture-word)	917 (99)	98 (2)	1017 (114)	89 (6)	911 (118)	99 (2)
Arithmetic Interference (operand-related-lure)	1266 (196)	94 (4)	1472 (246)	84 (9)	1332 (226)	94 (5)

*Note:* Mean response times (in milliseconds) and accuracy (in percent correct). Standard deviations are in parentheses.

**Table 2:** Brain regions associated with lexico-semantic and arithmetic interference.

	Brain Region	Cluster (%)	Extent	t-value	x	y	z
a) Arithmetic Interference (related > unrelated multiplication solutions)							
Left Hemisphere	L Insula Lobe	50.48	105	5.48	−30	25	8
	L IFG (p. Opercularis)	10.48					
	L IFG (p. Triangularis)	8.57					
	L Inferior Parietal Lobule (IPS)	12.04	108	5.17	−30	−58	45
	L Superior Parietal Lobule	7.41					
	L Superior Frontal Lobule	33.33	15	5.11	−28	−8	53
	L Middle Frontal Gyrus	13.33					
	L Precentral Gyrus	13.33					
	L IFG (p. Opercularis)	90.91	11	4.82	−55	10	35
	L Precentral Gyrus	9.09		4.86			
	L Cerebellum (Crus2)	77.78	9		−10	−75	−28
Right Hemisphere	R Insula Lobe	50.00	12	4.95	33	18	13
	R IFG (p. Triangularis)	54.55	11	5.01	40	30	23
	R Middle Frontal Gyrus	9.09					
	R Cerebellum 6	55.56	9	5.09	10	−75	−28
	R Cerebellum 6	50.00	2	4.78	30	−55	−28
b) Lexico-semantic Interference (related > unrelated picture-word pairs)							
Left Hemisphere	L Middle Frontal Gyrus	34.29	210	6.02	−40	13	33
	L IFG (p. Triangularis)	18.10					
	L IFG (p. Opercularis)	18.10					
	L Insula Lobe	7.78	90	6.26	−30	20	−8
	L IFG (p. Orbitalis)	33.33	57	5.35	−45	43	−10
	L Middle Frontal Gyrus (p. Orbitalis)	8.77					
	L Inferior Parietal Lobule (IPS)	3.85	26	5.21	−30	−55	45
	L Inferior Occipital Gyrus	5.56	18	5.10	−45	−73	−5
	L Caudate Nucleus	27.27	11	5.00	−13	3	13
	L Middle Temporal Gyrus	16.67	6	5.05	−60	−38	−10
	L Middle Occipital Gyrus	100.00	1	4.67	−28	−95	5
Right Hemisphere	R Medial part of the Sup. Front. Gyrus	60.45	440	7.17	0	33	45
	R Supplementary Motor Area	16.36					
	L Middle Cingulate Gyrus	13.18					
	R Inferior Occipital Gyrus	11.63	43	5.83	43	−65	−8
	R Inferior Temporal Gyrus	6.98					
	R Middle Occipital Gyrus	2.50	40	5.41	30	−93	5
	R IFG (p. Orbitalis)	16.67	30	4.95	35	20	−3
	R Insula Lobe	10.00					
	R Thalamus	58.33	24	5.13	3	−13	5
	L Thalamus	8.33					

*Notes:* Coordinates refer to the activation peak of the cluster and are reported in MNI (Montreal Neurological Institute) space as given by SPM12. The anatomical localization is presented based on the AAL (automated anatomical labeling) atlas (Tzourio-Mazoyer et al., 2002) and the SPM Anatomy toolbox (Eickhoff et al., 2005, 2006, 2007). The first label denotes the location of the peak activation, further labels indicate different brain regions within the same activation cluster (including submaxima) if the percentage of activated voxels within the cluster is > 5.00. Only activation clusters significant at *p* < .05 FWE corrected for multiple comparisons at peak level are reported. Abbreviations: L = left hemisphere; R = right hemisphere; IFG = inferior frontal gyrus; IPS = inferior frontal sulcus; p = pars.
