# Supplementary material for: Interference during the retrieval of arithmetic and lexico-semantic knowledge modulates similar brain regions: Evidence from functional magnetic resonance imaging (fMRI)
Source: Cortex. Author manuscript; Available in PMC 2019 Nov 13. (PMC6853793; doi:10.1016/j.cortex.2019.06.007)
Supplement: Appendix A [file EMS84658-supplement-Appendix_A.pdf]

Appendix A

Table A.1 – Experimental stimuli of the operand-related-lure task.

Answer Type	Solution Type	Run 1	Run 2	Run 3	Run 4
True	Correct	2 × 2 = 4	2 × 3 = 6	3 × 2 = 6	2 × 2 = 4
		2 × 4 = 8	2 × 5 = 10	5 × 2 = 10	4 × 2 = 8
		2 × 6 = 12	2 × 7 = 14	7 × 2 = 14	6 × 2 = 12
		2 × 8 = 16	2 × 9 = 18	9 × 2 = 18	8 × 2 = 16
		3 × 3 = 9	3 × 4 = 12	4 × 3 = 12	3 × 3 = 9
		3 × 5 = 15	3 × 6 = 18	6 × 3 = 18	5 × 3 = 15
		3 × 7 = 21	3 × 8 = 24	8 × 3 = 24	7 × 3 = 21
		3 × 9 = 27	4 × 4 = 16	4 × 4 = 16	9 × 3 = 27
		4 × 5 = 20	4 × 6 = 24	6 × 4 = 24	5 × 4 = 20
		4 × 7 = 28	4 × 8 = 32	8 × 4 = 32	7 × 4 = 28
		4 × 9 = 36	5 × 5 = 25	5 × 5 = 25	9 × 4 = 36
		5 × 6 = 30	5 × 7 = 35	7 × 5 = 35	6 × 5 = 30
		5 × 8 = 40	5 × 9 = 45	9 × 5 = 45	8 × 5 = 40
		6 × 6 = 36	6 × 7 = 42	7 × 6 = 42	6 × 6 = 36
		6 × 8 = 48	6 × 9 = 54	9 × 6 = 54	8 × 6 = 48
		7 × 7 = 49	7 × 8 = 56	8 × 7 = 56	7 × 7 = 49
		7 × 9 = 63	8 × 8 = 64	8 × 8 = 64	9 × 7 = 63
		8 × 9 = 72	9 × 9 = 81	9 × 9 = 81	9 × 8 = 72
False	Operand-related	3 × 2 = 9	2 × 2 = 6	2 × 4 = 6	2 × 5 = 15
		7 × 2 = 16	6 × 2 = 14	2 × 8 = 18	2 × 9 = 16
		4 × 3 = 15	3 × 3 = 12	3 × 5 = 20	3 × 6 = 21
		8 × 3 = 21	7 × 3 = 24	3 × 9 = 24	4 × 4 = 12
		6 × 4 = 30	5 × 4 = 25	4 × 7 = 24	4 × 8 = 28
		5 × 5 = 20	9 × 4 = 32	5 × 6 = 35	5 × 7 = 30
		9 × 5 = 40	8 × 5 = 45	6 × 6 = 30	6 × 7 = 48
		9 × 6 = 48	8 × 6 = 49	7 × 7 = 42	7 × 8 = 64
		8 × 8 = 56	9 × 7 = 64	8 × 9 = 81	9 × 9 = 72
	Operand-unrelated	5 × 2 = 17	4 × 2 = 7	2 × 2 = 5	2 × 3 = 7
		9 × 2 = 17	8 × 2 = 19	2 × 6 = 13	2 × 7 = 17
		6 × 3 = 22	5 × 3 = 22	3 × 3 = 11	3 × 4 = 13
		4 × 4 = 13	9 × 3 = 26	3 × 7 = 26	3 × 8 = 22
		8 × 4 = 26	7 × 4 = 26	4 × 5 = 22	4 × 6 = 34
		7 × 5 = 34	6 × 5 = 34	4 × 9 = 34	5 × 5 = 26
		7 × 6 = 46	6 × 6 = 34	5 × 8 = 44	5 × 9 = 44
		8 × 7 = 62	7 × 7 = 46	6 × 8 = 47	6 × 9 = 46
		9 × 9 = 74	9 × 8 = 82	7 × 9 = 62	8 × 8 = 52

Table A.2 – Experimental stimuli of the picture-word task.

Concept	Related Concept	Unrelated Concept
Hund (dog)	Katze (cat)	Stiefel (boot)
Kuh (cow)	Pferd (horse)	Glas (glass)
Löwe (lion)	Tiger (tiger)	Schloss (lock)
Schwan (swan)	Ente (duck)	Kamm (comb)
Adler (eagle)	Eule (owl)	Löffel (spoon)
Biene (bee)	Fliege (fly)	Schüssel (bowl)
Blume (flower)	Blatt (leaf)	Hut (hat)
Apfel (apple)	Birne (pear)	Schere (scissors)
Zange (pliers)	Schere (scissors)	Eule (owl)
Krone (crown)	Hut (hat)	Pferd (horse)
Kette (chain)	Schloss (lock)	Ente (duck)
Tasse (cup)	Glas (glass)	Katze (cat)
Pfanne (pan)	Schüssel (bowl)	Birne (pear)
Messer (knife)	Löffel (spoon)	Tiger (tiger)
Schuh (shoe)	Stiefel (boot)	Fliege (fly)
Bürste (brush)	Kamm (comb)	Blatt (leaf)
Katze (cat)	Hund (dog)	Tasse (cup)

(continued on next page)

Table A.2 – (*continued*)

Concept	Related Concept	Unrelated Concept
Pferd (horse)	Kuh (cow)	Krone (crown)
Tiger (tiger)	Löwe (lion)	Messer (knife)
Ente (duck)	Schwan (swan)	Kette (chain)
Eule (owl)	Adler (eagle)	Zange (pliers)
Fliege (fly)	Biene (bee)	Schuh (shoe)
Blatt (leaf)	Blume (flower)	Bürste (brush)
Birne (pear)	Apfel (apple)	Pfanne (pan)
Schere (scissors)	Zange (pliers)	Apfel (apple)
Hut (hat)	Krone (crown)	Blume (flower)
Schloss (lock)	Kette (chain)	Löwe (lion)
Glas (glass)	Tasse (cup)	Kuh (cow)
Schüssel (bowl)	Pfanne (pan)	Biene (bee)
Löffel (spoon)	Messer (knife)	Adler (eagle)
Stiefel (boot)	Schuh (shoe)	Hund (dog)
Kamm (comb)	Bürste (brush)	Schwan (swan)
